# Determination of the biological parameters of *Nabis pseudoferus orientarius* Remane, 1962 (Hemiptera: Nabidae) under laboratory conditions

**DOI:** 10.7717/peerj.20267

**Published:** 2026-01-13

**Authors:** Muhlis Sezgin, Selime Olmez Bayhan, Erol Bayhan

**Affiliations:** 1 Department of Entomology, Diyarbakir Plant Protection Research Institute, Diyarbakır, Turkey; 2 Faculty of Agriculture, Department of Plant Protection, Dicle (Tirgris) University, Diyarbakır, Turkey

**Keywords:** Life table, *Nabis pseudoferus orientarius*, Biological control, Side effect, Predator, Cotton

## Abstract

Cotton is an important industrial crop that is used in the textile and oil industries, in animal husbandry, and has economic value. Many insect species cause damage in cotton production areas. *Nabis* species are important predatory insects that feed on many soft-bodied insects, larvae, and eggs found in cotton fields. In this study, some biological parameters of *Nabis pseudoferus orientarius* were examined under laboratory conditions with 25 ± 1 °C temperature, 65 ± 5% humidity, and 16:8 L: D conditions. In the results of working, the average egg hatching time of *N. pseudoferus orientarius* was found with 9.83 days, the average preadult development time was 26.85 days, and the average adult life was 62.6 days. The average preoviposition, oviposition, and postoviposition periods of *N. pseudoferus orientarius* were determined as 9, 58.3, and 4.8 days, respectively. A female lays an average of 446.1 (267–576) eggs throughout her life. In the study, hereditary reproductive ability (r) was determined as 9.15, the increase rate limit (*λ*) was 1.10, the net reproductive rate (R_0_) was 193.13, and the average reproductive duration (T) was 57.50. The age-specific survival rate (l_x_) was calculated as 4.35, the age- and period-specific survival rate (S_xj_) was calculated as 80.30, and the age-specific reproduction rate (m_x_) was calculated as 14.

## Introduction

Cotton is an important industry in terms of providing employment opportunities to various people through its agricultural production and being used as a raw material source in the field of animal husbandry, especially in the textile sector, and in the seed and oil industry (Anonymous, 2018). India, China, the USA, Brazil, and Pakistan are seen as the top-producing countries in cotton production (Anonymous, 2022). Turkey is an important country in terms of cotton cultivation area, production amount, and fiber yield per unit area (Anonymous, 2019).

Cotton is a high-cost plant that requires intensive labor from planting to harvest. During the production period, some entomological problems occurred with many harmful insect species, causing serious losses in yield and quality. In recent years, interest in sustainable agricultural production and environmentally friendly practices has been increasing, and biological control methods have begun to attract more attention. Biological control involves the use of natural enemies against pests. Predatory insects are considered an effective factor to control pest populations in cotton production areas (Birişik et al., 2018).

Although the prevalence and density of predatory insects vary in cotton production areas, they are important biological control agents in the fight against pests. One of these is the Nabidae family, which has over 500 species in 20 genera (Faundez & Carvajal, 2014). The Nabidae family constitutes a group classified within the Hemiptera order, which is distributed worldwide. These organisms are divided into two subfamilies, Prostemmatinae and Nabinae, which exhibit marked differences in both morphology and ecology (Schuh & Slater, 1995). Nabidae are distinguished by their slender, elongated, and delicate body structure. Their colouration is typically light brown or grey. Their hunting behaviour has been observed to be associated with areas of plant cover (Henry, 2009). Nabidae species are defined as zoophagous (animal-eating) generalist predators. Research on their dietary preferences indicates that their feeding habits are predominantly influenced by the most prevalent prey species in their environment (Lattin, 1989). In addition to the “sit-and-wait” strategy, they actively search for prey. The consumption of prey by these organisms occurs *via* an external digestion process (extra-oral digestion), whereby prey are paralysed through the application of a sting by the rostrum, followed by the injection of digestive enzymes (Cohen, 1998). These are insects known for their long and soft bodies, which enable them to catch and hold their prey with their front legs. They are predators of many harmful insects in agricultural areas (Braman, 2000).

The Nabidae species of insect undergoes an incomplete metamorphosis, also known as hemimetabolism. This process includes five distinct nymphal stages, followed by the adult stage. The nymphs of the species under consideration are similar to their adult counterparts, except for the absence of wings. Their hunting behaviour is also comparable. Females lay their eggs in two different modes: firstly, individually, and secondly, in small groups within the plant tissues called parenchyma. The length of the egg incubation period depends on the ambient temperature. Most species undergo diapause, entering an inactive state that can last several years, in order to survive the winter. This is crucial for maintaining the population (Fauvel, 1999). Species belonging to the *Nabis* genus are slender, 0.5–1.0 cm long, and generally light grayish brown insects. These predators have a long, thin head and a stinging-sucking mouth structure. Their antennae are long and four-segmented, and they go through five nymph stages (Anonymous, 2020).

Insects hunted by Nabids consist mostly of small soft-bodied prey that feed on plants, red spiders, the *Lygus* genus, and lepidoptera eggs and larvae (Lattin, 1989). The efficacy of Nabidae as a biological control agent has been extensively documented in numerous field and laboratory studies. In laboratory studies on predation capacity, it has been demonstrated that a single adult *Nabis americoferus* can consume in excess of 50 *Lygus hesperus* Knight, 1971 (Hemipeta: Miridae) eggs or 10–15 aphids per day (Hagler, & Cohen, 1991). In addition, it has been established that species such as *Nabis pseudoferus* Remane, 1949 and *Nabis ferus* Linnaeus, 1758 (Hemiptera: Nabidae) play a pivotal role in the suppression of thrips and aphid populations in cotton, corn, and vegetable fields. The Nabidae are of particular value in integrated pest management programmes, owing to their extensive prey range. For instance, *Nabis pseudoferus* is a biological control agent that is mass-produced and released in greenhouses in the Mediterranean region against significant pests such as *Bemisia tabaci* Gannadius, 1889 (Hemiptera: Aleyrodidae) and *Frankliniella occidentalis* Pergande, 1895 (Thysanoptera: Thripidae) (Alomar, Goula, & Albajes, 2002). The Nabidae family is evidently a highly valuable and effective generalist predator for pest management in agricultural production. This is due to its broad prey spectrum, high prey consumption capacity, and ability to adapt to different agroecosystems. In particular, the conservation and promotion of species belonging to this family in sustainable and organic farming systems may be a key strategy in reducing the use of chemical insecticides.

Therefore, this makes the *Nabis* species important in cotton cultivation areas. To have information about the population, development, survival rates, and age distributions of natural enemies in biological control studies. When using natural enemies, the appropriate release time is important in determining the number of predators to be released and the number of releases. For this reason, in the fight against pests, bisexual life tables specific to age and period are important to determine the time of the fight (Atlıhan, Özgökçe & Chi, 2018).

The Şanlıurfa, Diyarbakır, and Mardin provinces in the Southeastern Anatolia Region are responsible for approximately 60% of cotton production in Turkey. In their seminal work, Sezgin & Ölmez Bayhan (2023) identified *Nabis pseudoferus orientarius* Remane, 1962 (Hemiptera: Nabidae) as a species of particular importance in cotton production areas. The objective of this study was to ascertain the biological parameters of *N. pseudoferus orientarius* that are significant in cotton production areas. These parameters were determined in a laboratory setting to elucidate the life history of this predatory insect. The findings pertaining to the hatching time, preadult developmental stages, adult lifespan, and productivity of *N. pseudoferus orientarius* will facilitate a more profound comprehension of the insect, offer insight into its biology in the wild, and contribute to the existing literature.

## Materials and Methods

### Growing Kalanchoe plants

*Kalanchoe blossfeldiana* plants that *Nabis pseudoferus orientarius* lays eggs on the petiole. It was grown in 21 L pots with a diameter of 40 cm and a height of 31 cm, using a $ \frac{1}{2} $ ratio of soil and peat mixture, in a fully automated greenhouse environment with a temperature of 24 ± 2 °C and humidity of 40 ± 5%. In the production of Kalanchoe plants, plant branches with 3–5 leaf eyes were cut and planted in pots during the study period, and plant continuity was ensured by propagation in this manner.

### Production of *Ephestia kuehniella*

Mass production of *Ephestia kuehniella* Zeller, 1879 (Lepidoptera: Pyralidae) was carried out according to Bulut & Kılınçer (1987) in a climate chamber with 25 ± 1 °C temperature and 65 ± 5% humidity, and 16:8 h light-dark conditions. In the production of *E. kuehniella*, 1/2 wheat flour and 1/2 coarse wheat bran, plastic tubs, containers covered with plastic tulle on both sides, and American cloth were used.

### Production of the *Nabis pseudoferus orientarius*

The specimens of the *Nabis* spp. (Hemiptera: Nabidae) utilised in the present study were collected in 2021 from cotton fields in the Gencan neighbourhood, Sur district, Diyarbakır province, employing the D-Vac sampling device. Adult and nymph individuals obtained from field studies were collected using a mouth aspirator and transferred to the laboratory in plastic containers (15 × 10 cm) ventilated with gauze cloth. The mass production of nymph and adult individuals was conducted within a Binder brand climate chamber. The production conditions were set at a temperature of 25 ± 1 °C, a relative humidity of 65 ± 5%, and a photoperiod of 16:8 h (light: dark). For the purpose of mass production, plastic containers measuring 15 × 10 cm and lined with gauze cloth were utilised, with each container accommodating a pair (consisting of one female and one male) of adult individuals. The experiment involved the addition of *Kalanchoe* sp. leaves to the containers, with the purpose of utilising them as an egg-laying substrate. The adult was fed using *Ephestia kuehniella* Zeller (Lepidoptera: Pyralidae) eggs, which were deposited on pieces of thin tracing paper (1 × 2 cm^2^).

Following the acquisition of a brood from the primary population, an experimental framework was established to ascertain the life history parameters. To this end, individuals from the newly emergent generation were utilised. The experiment was arranged in 30 replicates, with each replicate containing one newly emerged (0–24 h old) first-instar nymph. Each individual was regarded as a replicate and subjected to the same mass production conditions (25 ± 1 °C, 65 ± 5% RH, 16:8 L:D). The development times, survival rates, and reproductive parameters of the subjects were recorded on a daily basis.

### Determination of the biological parameters of *Nabis pseudoferus orientarius*

In the study, the primary population production of *N. pseudoferus orientarius* was conducted in 100 ml plastic sample containers covered with tulle, by feeding the eggs of *E. kuehniella* on 1 × 2 cm^2^ thin background paper. Adult individuals were placed in containers as one male and one female, and mass production was performed using the leaves of the Kalanchoe plant for laying eggs. After obtaining one progeny in the climate chamber, life table studies were initiated. In the daily controls over 30 eggs, the nymphs that hatched from the eggs were transferred one by one into plastic containers covered with gauze using a soft, fine-tipped brush, and their pre-adult development time was recorded. After the individuals reached adulthood, their sexes were determined based on the genital organs and abdominal structures of the adults, following the criteria described by Cornelis & Coscaron (2013), and the sex ratios were subsequently calculated. Then, the individuals of *N. pseudoferus orientarius*, one male and one female, were placed in plastic sample containers covered with tulle, and observations continued until they died.

In this study conducted under laboratory conditions, some biological characteristics of *N. pseudoferus orientarius*, such as egg hatching times, pre-adult development times, and mortality rates, preoviposition, oviposition, and postoviposition times of the adult female, adult lifespan, and sexual rates, were determined.

### Analysis of data

These data regarding the life period of *Nabis pseudoferus orientarius* were compiled using the TWOSEX-MSChart computer program (Chi, 1988) according to the age and period-specific twosex life chart developed by Chi (1988) and Chi & Liu (1985); Chi (1988). Calculated using Chi et al. (2022). Accordingly, life schedule parameters such as reproductive ability (r), increase rate limit (*λ*), net reproductive rate (R0), and average offspring duration (T), as well as lx (age-specific viability rate), S_xj_ (age- and period-specific viability). rate) and mx (age-specific reproductive rate) were calculated. The parameters in question were determined according to the formulas below (Özgökçe et al., 2021).

The age-specific survival rate “l_x_” value was calculated according to Eq. (2.2), and the reproductive rate “mx” values were calculated with the formula given in Eq. (2.1), where “*k*” is the number of periods, “S_xj_” is the survival of the egg at age x and period *j*. and “f_xj_” refers to the reproductive rate of adult individuals at age *x*.

(2.1) \begin{eqnarray*}{m}_{x}& = \frac{\sum _{j=1}^{k}{S}_{xj}{f}_{xj}}{\sum _{j=1}^{k}{S}_{xj}} \end{eqnarray*}

(2.2) \begin{eqnarray*}{l}_{x}& =\sum _{j=1}^{k}{S}_{xj}.\end{eqnarray*}

The age- and period-specific survival rate “S_xj_” value was calculated according to the formula in Eq. (2.3), “*x*” as age and “*j*” as period, “n_01_” as the total number of individuals used at the beginning of the study, “n_xj_” as *x* age, It represents the number of individuals alive in period *j*.

(2.3) \begin{eqnarray*}{S}_{xj}= \frac{{n}_{xj}}{{n}_{01}} .\end{eqnarray*}

Hereditary reproductive ability was determined as “r” x (age), l_x_ (age-specific viability rate), and m_x_ (age-specific reproductive power of the female individual) according to the formula given in Eq. (2.4) (Euler-Lotka (Goodman, 1982)),

(2.4) \begin{eqnarray*}\sum _{x=0}^{\infty }{e}^{-(x+1)}lxmx=1.\end{eqnarray*}

In the formula in Eq. (2.5) for the net reproductive rate “R_0_”, “l_x_” is defined as the rate of individuals surviving to *x* age, and “m_x_” is defined as the number of females produced per female of *x* age (Birch, 1948).

(2.5) \begin{eqnarray*}{R}_{0}=\sum _{x=0}^{\infty }{l}_{x}{m}_{x}.\end{eqnarray*}

The average generation time “T” refers to the duration needed to increase the size of the population by R0 times Eq. (2.6) (Birch, 1948; Carey, 1993).

(2.6) \begin{eqnarray*}\mathrm{T}= \frac{\ln \nolimits {R}_{0}}{r} .\end{eqnarray*}

The increase rate limit “*λ*” is expressed as the individual increase rate Eq. (2.7) per unit time (Birch, 1948).

(2.7) \begin{eqnarray*}\lambda ={e}^{2}.\end{eqnarray*}

## Results

Life statistical data of the developmental periods of *N. pseudoferus orientarius*, as daily checks and counts made in the studies of determining some biological characteristics of *N. pseudoferus orientarius* under laboratory conditions, are given in Table 1.

**Table 1:** Average data of the development periods of *Nabis pseudoferus orientarius* in the laboratory under 25 ± 1 °C temperature and 65 ± 5 percent humidity conditions (*n* = number of individuals).

Biological period	Female		Male		Pre-adult		Total	
	*n*	Mean ± SE	*n*	Mean ± SE	N	Mean ± SE	*n*	Mean ± SE
Egg	10	9.7 ± 0.21	10	10 ± 0	3	9.67 ± 0.33	23	9.83 ± 0.10
L1	10	3.2 ± 0.20	10	3.6 ± 0.22	3	2.33 ± 0.33	23	3.26 ± 0.16
L2	10	1.8 ± 0.25	10	1.7 ± 0.15	3	2.33 ± 0.33	23	1.83 ± 0.14
L3	10	2 ± 0.15	10	2.4 ± 0.22	3	2 ± 0	23	2.17 ± 0.12
L4	10	4.6 ± 0.40	10	3.6 ± 0.31	1	2 ± 0	21	4 ± 0.28
L5	10	5.1 ± 0.31	10	5 ± 0.26	–	–	20	5.05 ± 0.20
Adult	10	72.3 ± 2.48	10	53.4 ± 10.01	–	–	20	62.85 ± 5.47

According to the development periods of *N. pseudoferus orientarius* in Table 1, the average egg hatching period for female individuals was 9.7 days. The average nymph periods were 3.2, 1.8, 2.0, 4.6, and 5.1 days, respectively, while the adult period lasted 72.3 days. For male individuals, the average hatching period was 10 days, with nymph periods of 3.6, 1.7, 2.4, 3.6, and 5.0 days, respectively; the adult period was 53.4 days.

Average data regarding preoviposition, oviposition, and postoviposition times and the number of eggs laid by *N. pseudoferus orientarius* are given in Table 2.

**Table 2:** Preoviposition, oviposition, postoviposition times and number of eggs *of Nabis pseudoferus orientarius* under 25 ± 1 °C temperature and 65 ± 5 percent humidity conditions in the laboratory.

Parameter	Sex	*n*	Mean
Preoviposition time	*Female*	10	9.2
Oviposition time	*Female*	10	58.5
Postoviposition time	*Female*	10	4.6
Daily number of eggs	*Female*	10	6.18
Total number of eggs	*Female*	10	446.1

The preoviposition period of *N. pseudoferus orientarius* was 9.2 days on average, the average oviposition period was 58.5 days, and the postoviposition period was 4.6 days on average. When we look at egg production, a female gives an average of 446.1 eggs (267–576) throughout her life.

In this study, data on the life period of *N. pseudoferus orientarius* were obtained according to the age and period-specific bisexual life chart developed by Chi (1988) and Chi & Liu (1985) using the TWOSEX-MSChart computer program (Chi, 2020; Chi et al., 2022) and are given in Table 3. The average lifespan of *N. pseudoferus orientarius* was determined to be 80.30 days, and the fertility rate for females averaged 444.2 eggs.

**Table 3:** Lifetable data of *Nabis pseudoferus orientarius* under 25 ± 1 °C temperature and 65 ± 5 percent humidity conditions in the laboratory.

Parameter	Sex	*n*	Value
*The intrinsic rate of increase, r (day*^−1^)	*Female*	10	9.15
The net reproductive rate, R_0_ (female/female)	*Female*	10	193.13
*The finite rate of increase,λ(day*^−1^)	*Female*	10	1.10
Reproductive rate, F (female/female)	*Female*	10	444.2
Average fertilization period, T (day)	*Female*	10	57.50
Population doubling time, DT (day)	*Female*	10	7.58

According to the life period of *N. pseudoferus orientarius*, hereditary reproductive ability (*r*) is 9.15 (day^−1^), the increase rate limit (*λ*) is 1.10 (day^−1^), net reproduction rate (R_0_) is 193.13 (female/female), and the average progeny. The period (T) was determined with 57.50 days, and the population doubling time (DT) was determined with 7.58 days. Unlike the data obtained in this study, Efe & Karaca (2013) included the following parameters in the life table of *N. pseudoferus*; reported hereditary reproductive ability (*r*_*m*_) as 0.079 female/female/day, net reproductive power (R_0_) as 31,000 females/female/day, mean generation time (T_0_) as 43,246 days, and increase rate limit (*λ*) as 1.083. It is thought that the reason for the difference between the values obtained from these studies may be the use of different nutrients and analysis methods.

**Figure 1:**
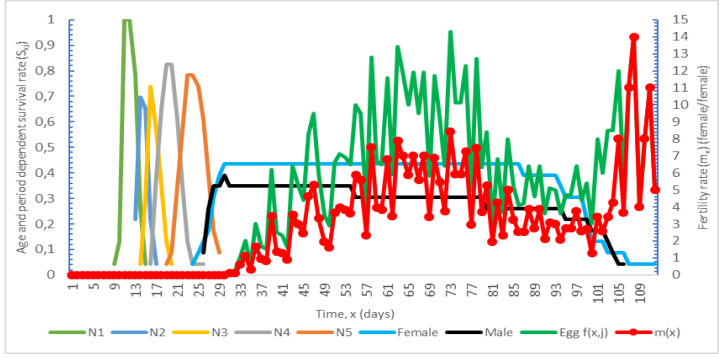
Age and period-dependent survival rate (S_xj_) and fertility (m_x_) graph of *Nabis pseudoferus orientarius*.

Age and period-dependent survival rate (*S*_*xj*_) and fertility (*m*_*x*_) graphs of *Nabis pseudoferus orientarius*. It is given in Fig. 1.

The age- and period-dependent survival rate (*S*_*xj*_) was calculated as 80.30, and the age-specific reproductive rate (*m*_*x*_) was calculated as 14 Chi & Liu (1985).

As a result, in this study, the average hatching time of *N. pseudoferus orientarius* nymphs was determined as 9.8 days, the average preadult development period was 26.35 days, the average adult lifespan was 62.6 days, and the average oviposition period of *N. pseudoferus orientarius* was 58.3 days. A female lays an average of 446.1 (267–576) eggs throughout her life. In the study, hereditary reproductive ability (*r*) was 9.15, the increase rate limit (*λ*) was 1.10, the net reproductive rate (R_0_) was 193.13, and the average reproductive duration (T) was 57.50.

## Discussion

Similarly, in a study conducted by Perkins & Watson (1972) on *N. alternatus* regarding the preadult development periods for male individuals, it was reported that the 1st nymph period averaged 2–5 days, the 2nd nymph period averaged 1–5 days, the 3rd nymph period averaged 1–7 days, the 4th nymph period averaged 2–6 days, and the 5th nymph period averaged 3–7 days. For female individuals, the average preadult development periods were: 1st nymph period at 1–7 days, 2nd nymph period at 1–5 days, 3rd nymph period at 1–7 days, 4th nymph period at 2–7 days, and 5th nymph period at 3 days. They found it to be -8 days. However, Efe & Karaca (2013) examined the life parameters of *Spodoptera littoralis*; the egg hatching period for *N. pseudoferus* was 12.85 ± 0.23 (11–14) days, the 1st nymph period was 3.18 ± 0.17 (2–7) days, the 2nd nymph period was 2.64 ± 0.15 (2–7) days, and the 3rd nymph period was 2.64 ± 0.15 (2–7) days. They determined that the nymph period was 2.72 ± 0.09 (2−3.66) days, the 4th nymph period was 2.82 ± 0.17 (1−4.29) days, and the 5th nymph period was 6.43 ± 0.15 (5−7.62) days. There are differences between the mean values in Table 1 for the preadult developmental stages of *N. pseudoferus orientarius* and the results of the previous study. These differences may be due to the use of different nutrients in these two studies.

Perkins & Watson (1972) reported the preoviposition period for *Nabis alternatus* as 4–16 days, the oviposition period as 3–65 days, the postoviposition period as 0–27 days, and the total number of eggs laid as 17–595. In this study, the average preoviposition, oviposition, and postoviposition times of *N. pseudoferus orientarius* were within the value ranges given for *Nabis alternatus* in the previous study, and both species showed similar results in terms of the highest egg production. Efe & Karaca (2013) reported the oviposition period of *N. pseudoferus* as 25.31 (8.00–53.00) days and the postoviposition period as 30.26 (1.00–75.00) days. The oviposition and postoviposition time values of *N. pseudoferus orientarius* differed from those given in the Efe & Karaca (2013) study. It is thought that these different results may be due to the different foods used in the studies.

## Conclusion

This study provides a comprehensive analysis of the life history parameters of *N. pseudoferus orientarius*. Key developmental periods and reproductive characteristics were meticulously documented for both female and male individuals. The findings of the present study indicate that the average egg hatching period was consistent across sexes, with females averaging 9.7 days and males 10 days. A significant disparity was observed in the adult lifespan, with females exhibiting a substantially longer lifespan (72.3 days) in comparison to males (53.4 days). The mean nymph periods, while exhibiting variation across instars, contributed to an overall mean lifespan of 80.30 days for *N. pseudoferus orientarius*. A comprehensive investigation was conducted into the reproductive parameters of the female subjects. The preoviposition period exhibited an average duration of 9.2 days, succeeded by an extended oviposition period of 58.5 days. The postoviposition period was comparatively brief, with an average duration of 4.6 days. It is noteworthy that the mean fertility rate for females was determined to be 444.2 eggs, with a mean lifetime egg production averaging 446.1 eggs, ranging from 267 to 576. The calculations pertaining to population dynamics have yielded the following results: the hereditary reproductive ability (*r*) has been established at 9.15 (day^−1^), and the increase rate limit (*λ*) has been determined to be 1.10 (day^−1^). The net reproduction rate (R) is a key metric in demography, measuring the rate at which the population is growing or declining. The number zero is a numeral used to denote the absence of quantity, and in this context, it is employed to signify the absence of the specified element. The following text is intended to provide a comprehensive overview of the subject matter. The female-to-male ratio was found to be high at 193.13, indicating robust population growth. The mean progeny period (T) was thus determined to be 57.50 days, and the population doubling time (DT) was found to be 7.58 days.

##  Supplemental Information

10.7717/peerj.20267/supp-1 Supplemental Information 1 Life table output

## Additional Information and Declarations

### Data Availability

The following information was supplied regarding data availability: The raw data is available in the Supplementary File.
